# Patterns and persistence of SARS-CoV-2 IgG antibodies in Chicago to monitor COVID-19 exposure

**DOI:** 10.1172/jci.insight.146148

**Published:** 2021-05-10

**Authors:** Alexis R. Demonbreun, Thomas W. McDade, Lorenzo Pesce, Lauren A. Vaught, Nina L. Reiser, Elena Bogdanovic, Matthew P. Velez, Ryan R. Hsieh, Lacy M. Simons, Rana Saber, Daniel T. Ryan, Michael G. Ison, Judd F. Hultquist, John T. Wilkins, Richard T. D’Aquila, Brian Mustanski, Elizabeth M. McNally

**Affiliations:** 1 Center for Genetic Medicine and; 2 Department of Pharmacology, Northwestern University Feinberg School of Medicine, Chicago, Illinois, USA.; 3 Department of Anthropology and Institute for Policy Research, Northwestern University, Evanston, Illinois, USA.; 4 Division of Cardiology, Department of Medicine, and; 5 Division of Infectious Diseases, Department of Medicine, Northwestern University Feinberg School of Medicine, Chicago, Illinois, USA.; 6 Institute for Sexual and Gender Minority Health and Wellbeing and Department of Medical Social Sciences, Northwestern University, Chicago, Illinois, USA.; 7 Division of Organ Transplantation, Department of Surgery, and; 8 Department of Preventive Medicine, Northwestern University Feinberg School of Medicine, Chicago, Illinois, USA.; 9 Department of Biochemistry and Molecular Genetics, Northwestern University, Chicago, Illinois, USA.

**Keywords:** COVID-19, Immunoglobulins

## Abstract

**BACKGROUND:**

Estimates of seroprevalence to SARS-CoV-2 vary widely and may influence vaccination response. We ascertained IgG levels across a single US metropolitan site, Chicago, from June 2020 through December 2020.

**METHODS:**

Participants (*n* = 7935) were recruited through electronic advertising and received materials for a self-sampled dried-blood spot assay through the mail or a minimal contact in-person method. IgG against the receptor-binding domain of SARS-CoV-2 was measured using an established highly sensitive and highly specific assay.

**RESULTS:**

Overall seroprevalence was 17.9%, with no significant difference between method of contact. Only 2.5% of participants reported having had a diagnosis of COVID-19 based on virus detection, consistent with a 7-fold greater exposure to SARS-CoV-2 measured by serology than that detected by viral testing. The range of IgG level observed in seropositive participants from this community survey overlapped with the range of IgG levels associated with COVID-19 cases having a documented positive PCR test. From a subset of those who participated in repeat testing, half of seropositive individuals retained detectable antibodies for 3 to 4 months.

**CONCLUSION:**

Quantitative IgG measurements with a highly specific and sensitive assay indicated more widespread exposure to SARS-CoV-2 than observed by viral testing. The range of IgG concentrations produced from these asymptomatic exposures was similar to IgG levels occurring after documented nonhospitalized COVID-19, which were considerably lower than those produced from hospitalized COVID-19 cases. The differing ranges of IgG response, coupled with the rate of decay of antibodies, may influence response to subsequent viral exposure and vaccine.

**Funding:**

National Science Foundation grant 2035114, NIH grant 3UL1TR001422-06S4, NIH National Center for Advancing Translational Sciences grants UL1 TR001422 and UL1 TR002389, Dixon Family Foundation, Northwestern University Cancer Center (NIH grant P30 CA060553), and Walder Foundation’s Chicago Coronavirus Assessment Network.

## Graphical abstract

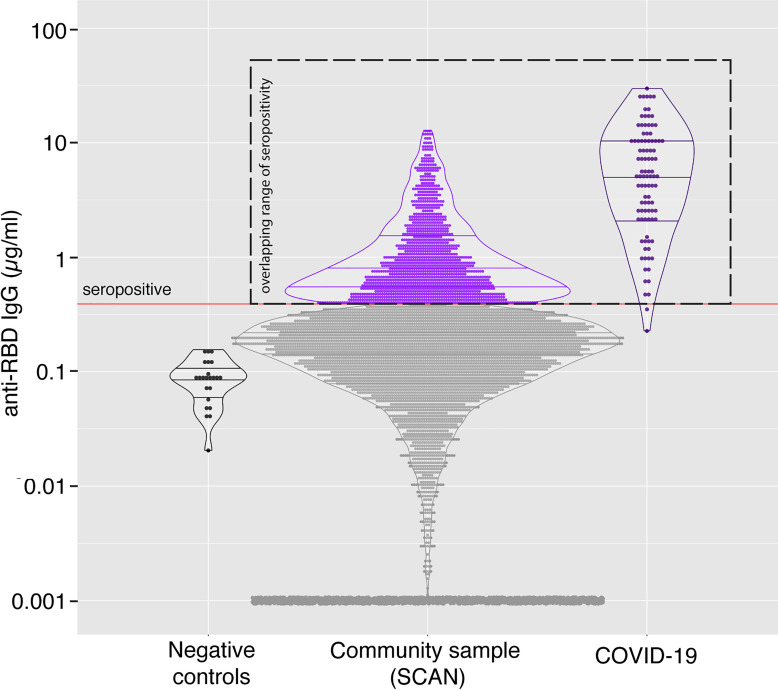

## Introduction

The presence of serum antibodies specific to SARS-CoV-2, the virus that causes COVID-19, reflects prior exposure. Seroprevalence, or seropositivity, estimates range from 3%–50% depending on the population surveyed, method of testing, and viral antigen target (1–13). Most tests measure antibody content in blood or serum, which often necessitates contact with a health care facility. Point-of-care lateral flow devices have also been employed but lack both the sensitivity and specificity of a laboratory-performed measurement (3, 14).

As an alternative, dried-blood spots (DBS) can be easily collected at home using a simple finger prick method, and then the DBS can be used in a laboratory-performed assay to yield quantitative IgG measurements. We used DBS in an assay that indexes IgG levels to an antibody with known affinity to yield a concentration in micrograms per ml (μg/ml). The antigen in this assay is restricted to the receptor-binding domain (RBD), a small and specific domain within the SARS-CoV-2 spike protein; multiple studies have documented specificity of 97.7%–100% when using this target, owing to RBD’s limited sequence homology to other viruses (1, 15, 16). DBS sampling was performed on nearly 8000 participants across Chicago. Approximately half of the participants were recruited from a wide socioeconomic range by zip codes. These participants received and returned their DBS test kits through the US Postal Service. The remaining participants were primarily non-healthcare-providing students, staff, and faculty affiliated with a medical school who received and returned DBS kits after having minimal contact with the study staff.

The total cohort included a mix of individuals who were working outside the home as well as those working inside the home during the testing interval. The sampling began in late June 2020, in a period when local shelter-in-place orders were partially relaxed, and continued through December 2020. Overall, the IgG seroprevalence was 17.9%, with similar seroprevalence among samples ascertained through mail and those obtained through on-site DBS kit distribution. Moreover, seroprevalence did not differ between those working inside and those working outside the home. The IgG range of seropositive individuals overlapped with that of documented, nonhospitalized COVID-19 cases, consistent with widespread exposure to COVID-19 through the second half of 2020.

## Results

### Prevalence of IgG antibodies against the receptor-binding domain of SARS-CoV-2.

**Figure 1:**
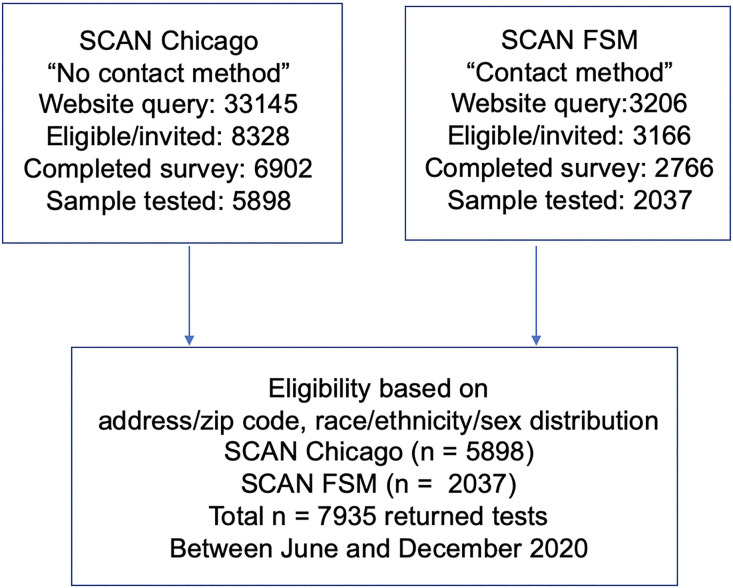
Flow diagram for recruitment into Screening for Coronavirus Antibodies in Neighborhoods studies. Participants were recruited to enter queries to the Screening for Coronavirus Antibodies in Neighborhoods (SCAN) website through social media, news coverage, and paid advertising with focus on zip codes throughout Chicago. Individuals were screened for eligibility based on living in specific zip codes and recruited to promote a racially/ethnically mixed cohort, with adequate representation of men and women, and then invited to complete a health questionnaire survey. Dried-blood spot kits were sent to all eligible participants who completed the survey. These participants received and returned dried-blood spot kits through the mail (no contact method) with an 85% return rate. A second cohort was recruited by email through the Northwestern’s Feinberg School of Medicine (FSM), and these individuals received blood spot kits in person and returned kits on site (contact method) with a 74% return rate.

The Screening for Coronavirus Antibodies in Neighborhood (SCAN) study uses an at-home testing strategy to measure IgG antibodies against the receptor-binding domain (RBD) of the SARS-CoV-2 spike protein (16). IgG assays with RBD as antigen have shown 97.7%–100% specificity and 81%–89% sensitivity (15–17). Participants received a kit to provide a DBS sample from a finger prick, and DBS cards were used in a quantitative laboratory-performed ELISA. Between June and December 2020, 7935 SCAN samples were collected. Of SCAN participants, 195 (2.5%) reported having COVID-19 from a prior positive diagnostic test for SARS-CoV-2 virus. Participants (*n* = 5898) were recruited through advertising and social media, and participants received and returned test materials through the mail (no-contact method) (Figure 1). An additional 2037 were solicited through email and in-person contact to provide and retrieve the DBS materials (contact method). Seropositivity in SCAN participants utilizing the no-contact method was 18.2 % (*n* = 1072 of 5898), while the seropositivity among the group who used the contact method was 17.3 % (*n* = 352 of 2037) (odds ratio 1.06; *P* = 0.4; CI 0.93–1.23).

Of the total 7935 SCAN participants, 195 (2.5%) reported having COVID-19 with a prior positive virus test, with 169 of 195 (86.6%) seropositive for RBD IgG. In the total cohort of 7935 participants, 1424 (17.9%) were seropositive and 6511(82.1%) were seronegative. This represents 7 times more seropositive samples than confirmed by reports of SARS-CoV-2 nucleic acid positivity in the SCAN cohort. Seropositivity was similar between men at 18.8% (615 of 3278) and women at 17.4% (809 of 4657) (Table 1). Seropositivity by age group varied slightly from 20.9% (18–29 years), 17.2 % (30–39 years), 17.6% (40–49 years), 18.0% (50–59 years), and 14.0% (60+ years) (Table 2).

### IgG serum levels in SCAN overlap with IgG levels in outpatient COVID-19 cases.

**Figure 2:**
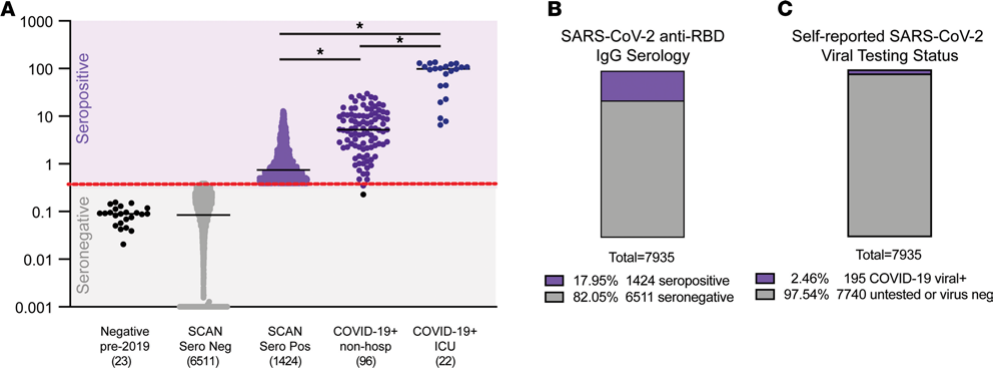
Quantitative measure of IgG directed to the receptor-binding domain of SARS-CoV-2 spike glycoprotein. Samples were acquired through Screening for Coronavirus Antibodies in Neighborhood (SCAN) between June 2020 and December 2020 (*n* = 7935). (**A**) Samples acquired before 2019 constituted the negative, pre-COVID group (leftmost column; black dots; median, 0.09 μg/ml), and the mean IgG was similar to that in the SCAN seronegative group (second column; gray dots; median, 0.09 μg/ml). The median IgG range in SCAN seropositive samples (middle column; light purple) was 0.75 μg/ml. The median IgG range in outpatient, nonhospitalized COVID-19 samples (4th column, dark purple) was 5.2 μg/ml. The range between SCAN seropositive and outpatient COVID-19 samples showed significant overlap. Both SCAN seropositive and outpatient COVID-19 cases had much lower IgG levels than ICU-hospitalized COVID-19 cases (rightmost column, dark blue, median 98.5 μg/ml). The SARS-CoV-2 receptor-binding domain (RBD) IgG ELISA seropositive threshold is marked by the red line at 0.39 μg/ml, as validated previously (16). *P < 0.0001, comparing seropositive groups, by Wilcoxon-Mann-Whitney test. Both true negatives and the SCAN seronegative groups were significantly different compared with all seropositive groups. (**B**) 17.95% (1424 of 7935) of SCAN samples were seropositive. (**C**) 2.46% (195 of 7935) reported having a positive SARS-CoV-2 viral diagnostic test.

The CR3022 antibody has known affinity for the target antigen, making it possible to quantify IgG directed at RBD. There was no difference in the mean IgG level in seronegative samples compared with that in samples collected in 2018 (pre-COVID-19 samples) (median 0.09 μg/ml vs. 0.09 μg/ml). The median IgG concentration from seropositive samples from SCAN was 0.75 μg/ml. As a comparison, the median IgG from those with a COVID-19 diagnosis based on virus detection (*n* = 96), but who did not require hospitalization, was 5.2 μg/ml, while the median IgG from those requiring ICU hospitalization was 98.5 μg/ml (Figure 2). While the median levels differ between documented COVID-19 cases and SCAN seropositive participants, the range of IgG showed wide overlap between these groups, indicating extensive range in IgG response among those with outpatient COVID-19 and those with asymptomatic exposure.

### Similar levels of SARS-CoV-2 RBD IgG antibodies in essential and nonessential workers.

**Figure 3:**
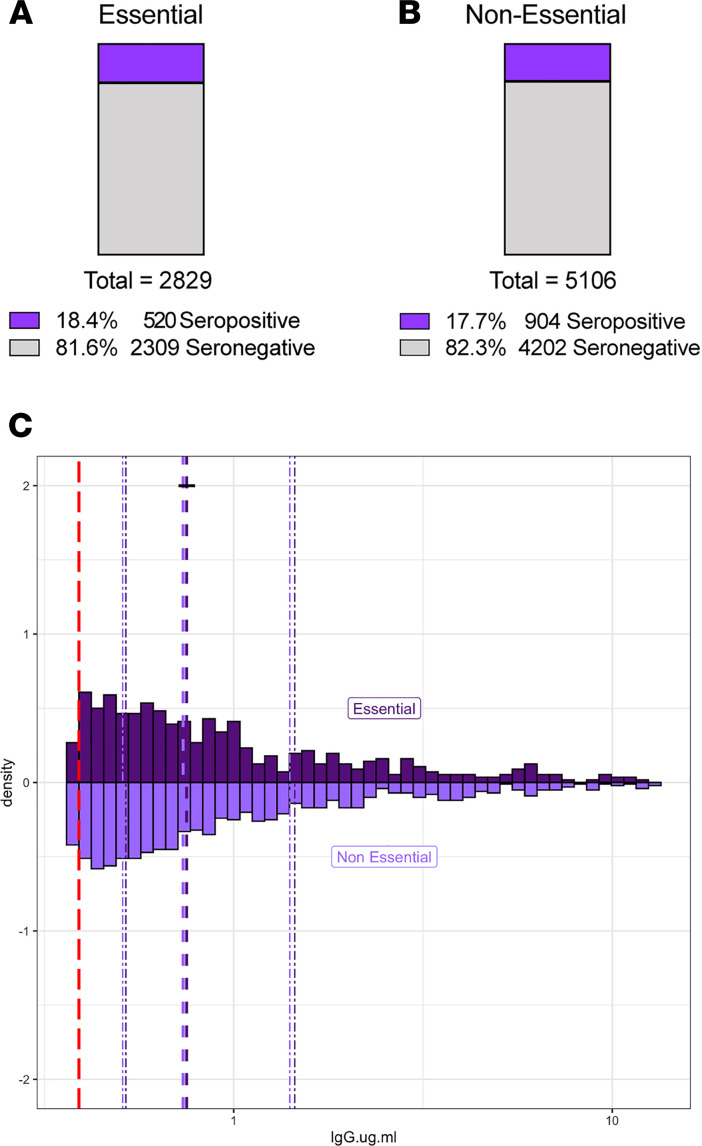
Similar rates of SARS-CoV-2 RBD IgG seropositivity between those who reported working outside the home and those working from home. 7935 unique SCAN community-acquired samples were acquired between June and December 2020 from the Chicago area. Participants self-reported whether they left their residence for work (essential) and interacted with coworkers/public. (**A** and **B**) Reported groups of essential workers and those working from home (nonessential) have similar percent seropositivity, at 18.4 % and 17.7%, respectively. (**C**) Essential (*n* = 520) and nonessential (*n* = 904) groups had similar distributions of SARS-CoV-2 RBD IgG seropositivity, with a median of 0.75 μg/ml and 0.74 μg/ml, respectively. The SARS-CoV-2 RBD IgG ELISA positivity threshold is denoted with the red dotted line at 0.39 μg/ml. Dashed purple lines represent quartiles. *P* = 0.86, 2-sample Kolmogorov-Smirnov test.

Between March 21 and May 30, 2020, the state of Illinois and the city of Chicago were under a shelter-in-place order, except for certain workers and essential trips. Between June and August 2020, these orders were gradually relaxed, but limits on gatherings of more than 50 people and restrictions on indoor dining/bars remained in effect. In late November 2020, a stay-at-home advisory was put in place in the city of Chicago asking residents to only leave home for essential activities, reflecting the earlier second wave experienced in the Midwest. SCAN participants reported whether they left their place of residence and interacted with others at the workplace during the shelter-in-place or stay-at home intervals. Essential (*n* = 2829) and nonessential (*n* = 5106) groups had similar a percentage of seropositivity at 18.4% and 17.7%, respectively (Figure 3, A and B). The two groups were well matched by design for age, sex, and race (Table 3). The median RBD IgG level and distribution were not different (*P* = 0.86) between the seropositive essential (*n* = 520, median 0.75 μg/ml) and seropositive nonessential groups (*n* = 904, median 0.74 μg/ml) (Figure 3C).

### Only modest agreement between RBD and nucleocapsid serology status.

**Figure 4:**
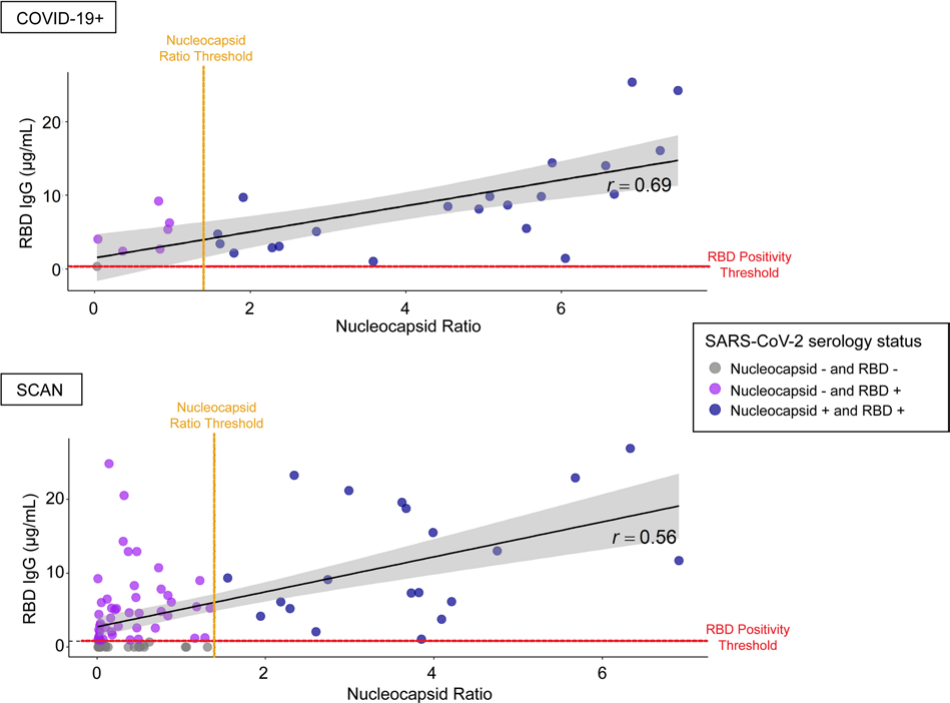
Modest agreement between SARS-CoV-2 RBD IgG and nucleocapsid seropositivity. Twenty-eight COVID-19^+^ viral, nonhospitalized samples and 92 SCAN samples with untested/negative COVID-19 status were analyzed for the presence of SARS-CoV-2 RBD IgG and nucleocapsid IgG antibodies using a hospital performed test. Six of twenty-eight (21.5%) known COVID-19^+^ viral samples were nucleocapsid^–^ but were RBD IgG^+^. One of twenty-eight COVID-19^+^ viral samples was seronegative on both platforms. Of the 92 unknown COVID-19 status samples, 20 (21.7%) samples were both nucleocapsid and RBD IgG^+^, while 45 (48.9%) samples were nucleocapsid^–^ and RBD IgG^+^. Agreement between RBD IgG and nucleocapsid classification was modest for both the known COVID-19^+^ viral (κ = 0.20; 95% CI, 0.00–0.59) and unknown COVID-19 status (κ = 0.21; 95% CI, 0.12–0.32) samples. The black diagonal line and gray shaded area represent the simple linear regression of RBD IgG on nucleocapsid ratio and the 95% CI band, respectively. The SARS-CoV-2 RBD IgG ELISA positivity threshold is denoted with the red dotted line at 0.39 μg/ml. The SARS-CoV-2 nucleocapsid positivity threshold is denoted with the orange dotted line at ratio 1.4.

We assayed 28 samples from individuals who recovered from symptomatic COVID-19 with positive virus diagnostic testing and 92 suspected samples from individuals who did not report a positive virus diagnostic test result. Agreement between RBD IgG and nucleocapsid classification was modest for both the COVID-19^+^ (Cohen’s κ coefficient, κ = 0.20; 95% CI, 0.00–0.59) and suspected SCAN group (κ = 0.21; 95% CI, 0.12–0.32) groups (Figure 4). Specifically, 6 of 28 (21.4%) COVID-19^+^ samples had no IgG against nucleocapsid using a hospital-performed, FDA-authorized assay. Only 1 of these 28 samples was below the limit of detection in the RBD assay, and this same sample was also negative in the nucleocapsid assay. The remaining 27 of 28 were positive for RBD IgG. A similar analysis of 92 suspected positive SCAN samples was conducted; this cohort was selected to skew toward seropositivity based on reported possible exposure or mild symptoms. Of the 92 “suspected” samples, 65 (70.7%) samples had IgG against RBD. Of these 65 positive samples, 20 samples had IgG against both RBD and nucleocapsid, while the remaining 45 had only IgG against RBD and not to nucleocapsid. In both groups, there were no samples that were RBD^–^ and nucleocapsid^+^. These data suggest limited sensitivity of nucleocapsid testing.

### Persistence of SARS-CoV-2 RBD IgG antibodies over time.

**Figure 5:**
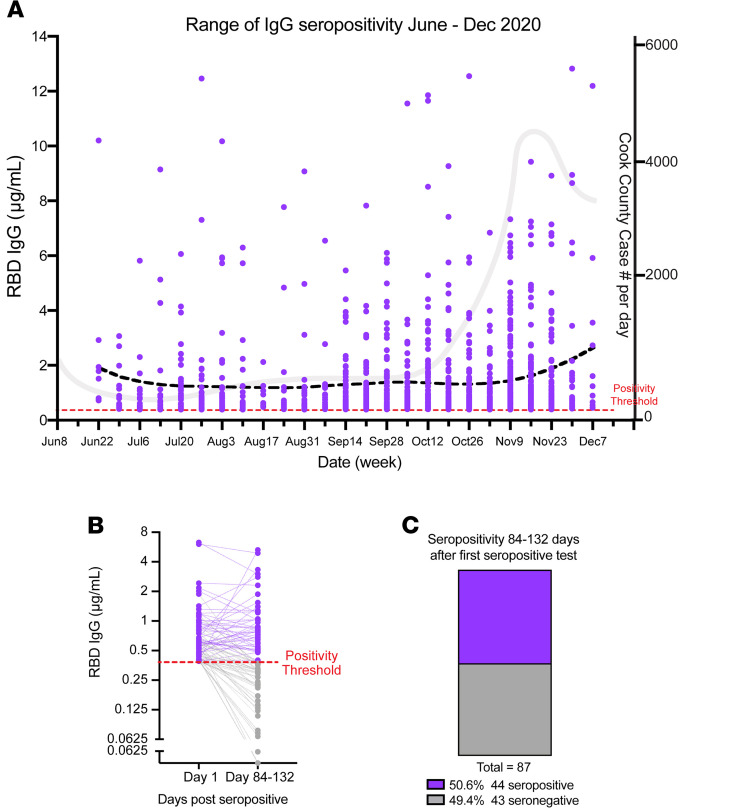
Detectable SARS-CoV-2 RBD IgG antibodies after 3–4 months in SCAN community samples. (**A**) Two hundred and eighty-six seropositive RBD IgG SCAN sample concentrations plotted as a function of calendar week of acquisition. The Lowess curve (black dotted line) is steady across 26 weeks of sampling. The gray line illustrates the COVID case number per day in Cook County. (**B** and **C**) Eighty-seven seropositive SCAN participants (purple dots) were resampled 84–132 days after the first seropositive RBD IgG test (mean 103 days). Dotted lines connect the same participant over time. Eighteen of eighty-seven (50.6%) samples remained seropositive after 3–4 months, with 43 (49.4%) samples converting to seronegative (gray dots). Time points were significantly different (*P* < 0.0001 by Wilcoxon signed rank test).

We monitored change in RBD IgG concentration across the cohort of seropositive SCAN participants (*n* = 1305) over time. IgG concentration in a cross-sectional comparison at each time point remained similar over 26 weeks from June to December 2020 (Figure 5A). We separately assessed longevity of IgG persistence in 87 seropositive participants with repeat sampling 3–4 months (mean 103 days) from their first detected seropositivity (Figure 5B). The median IgG concentration at day 0, the first day of seropositivity, was 0.59 μg/ml, above the 0.39 μg/ml positivity threshold. After 84–132 days, the median IgG concentration decreased to 0.397 μg/ml (*P* < 0.001). Forty-four of eighty-seven seropositive samples (50.6%) remained seropositive over 84–132 days after the first seropositive result (Figure 5C). Of those remaining seropositive samples, 24 of 44 (54.5%) had similar or increased RBD IgG levels upon resampling, while 20 of 44 (45.5%) had decreased RBD IgG concentration. These data show that half of SCAN participants who were seropositive at their first test had detectable IgG antibodies against SARS-CoV-2 RBD at 3–4 months.

**Figure 6:**
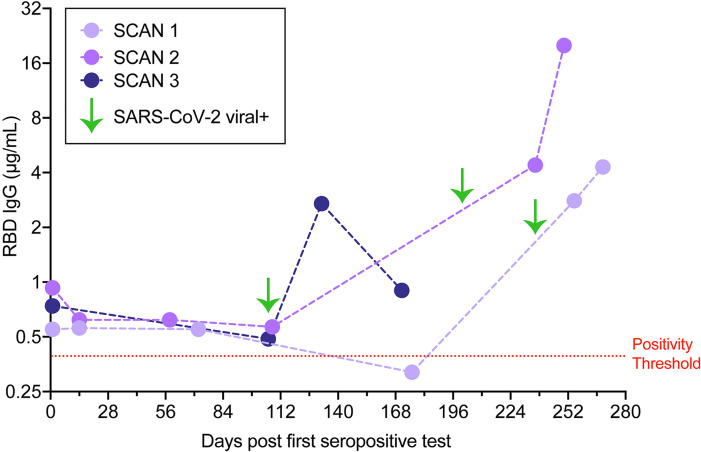
Reexposure to SARS-CoV-2. Three IgG RBD seropositive individuals (SCAN 1, 2, 3) were observed to have a marked increase in IgG against RBD 14–28 days after positive SARS-CoV-2 diagnostic viral testing (green arrows). Mild symptoms were noted, including cough, altered taste, and smell. None required hospitalization.

Three individuals with stable seropositive RBD IgG levels, over at least a 3-month period, reported subsequently having a positive SARS-CoV-2 diagnostic virus test result despite the presence of antibodies against RBD. Each reported having COVID-19 symptoms, and none required hospitalization. The participants sought testing for COVID-19 after experiencing several days of cough or altered taste and smell. RBD IgG levels 14–28 days after the positive SARS-CoV-2 diagnostic test increased in all 3 individuals (Figure 6). This seroprevalence pattern is likely to reflect reexposure to SARS-CoV-2, confirmed by a viral diagnostic result at the second exposure, and demonstrates a strong immune response after a second exposure.

## Discussion

### Estimating SARS-CoV-2 exposure with quantitative determination of RBD antibodies.

We surveyed the presence of IgG against a restricted region of the spike glycoprotein, as this domain has been shown to be highly specific to SARS-CoV-2 (17). The 17.9% seropositivity rate in this study is consistent with this antibody test identifying 7-fold greater exposure than noted with virus diagnostic testing, supporting previous predictions (18). The median level of IgG among seropositive cases was lower than that among documented COVID-19 outpatient cases, but the range of IgG overlapped between these two groups. RBD IgG levels were much higher in ICU-hospitalized COVID-19 cases. It is the RBD portion of the spike protein that mediates viral entry into cells, and some antibodies against the RBD can be neutralizing and protect against cellular invasion. Whether the RBD antibodies detected in the SCAN study and mildly symptomatic or asymptomatic COVID-19 are neutralizing or protective in nature is unknown. It also remains to be determined whether or how much these lower levels of RBD IgG serve to prime the immune system on reexposure or for subsequent vaccination.

### RBD versus nucleocapsid antibodies.

We found that among individuals with documented symptomatic COVID-19, there was a lower detection rate for the nucleocapsid antigen, using a hospital-performed, FDA-authorized test. The discordance between anti-nucleocapsid and anti-RBD IgG antibody detection was noted in a study of COVID-19^+^ serum using other independent platforms (Pearson’s correlation of 0.65) (19). Among RBD IgG seropositive individuals that were asymptomatic or mildly symptomatic, there was even less concordance between anti-nucleocapsid and anti-RBD IgG antibody detection. Gudbjartsson et al. reported similar findings in an Icelandic cohort of individuals untested or negative for SARS-CoV-2, where only 44.3% of anti-RBD IgG^+^ samples were also positive for anti-nucleocapsid IgG antibody (5). This differential sensitivity may represent differences in antibody response, testing platform, or both. Similar to others, we favor the use of RBD IgG determination, as this affords high specificity given the unique nature of the target domain.

Testing for COVID-19 virus became much more widely available beginning in May and June 2020 and increased exponentially thereafter, yet only 2.5% of individuals in this survey reported a positive COVID-19 test consistent with a high rate of asymptomatic exposure to virus across many areas of Chicago. Although the SCAN cohort derives from a wide range of Chicago neighborhoods, the cohort could be biased by those self-enrolling due to higher concerns for SARS-CoV-2 exposure risk.

### Persistence of IgG directed to RBD.

During the summer of 2020, local restrictions imposed during spring 2020 were eased. This included allowing retail stores to open, outdoor dining, larger gatherings, and gym reopening. Mitigation measures were then reinstated in November 2020 related to the early second wave that spread across the Midwest and Chicago during October. We observed a similar seroprevalence among participants who self-reported as leaving the home for work during the restricted periods and those who remained at home. We observed 3 cases of documented SARS-CoV-2 viral infection in individuals with prior seropositivity, and each developed a strong “boost” of IgG against RBD after having documented viral infection. We expect that this pattern reflects what occurs with second exposure and potentially mimics what might occur with vaccination. The SCAN platform, which relies on simple at-home monitoring combined with laboratory precision, is positioned to help address this and other knowledge gaps.

## Methods

### Study recruitment.

Participants were recruited through two mechanisms. Community-based participants were recruited from 10 zip codes in Chicago through social media advertising and news articles. Alternatively, staff, students, and faculty from Northwestern University Feinberg School of Medicine were sent an email describing the study, with a link to the website. Participants were screened for eligibility (zip code and demographics or affiliation to Northwestern). Eligible participants were then invited to complete a questionnaire regarding health status, including COVID-19 symptoms. Community participants received materials for DBS collection through the USPS and returned their test kits using prepaid USPS envelopes provided to them by the study team. Those affiliated with Northwestern were given a specific time to collect DBS kits in person and were instructed to return their completed kits to a secure unmanned collection box. Sample collection occurred between June and December 2020. Eighty-seven participants were resampled 84–132 days after the first seropositive RBD IgG test. Samples from 2018 (*n* = 23) were used as negative controls. Comparator samples derived from 40 COVID-19 nonhospitalized cases, 22 hospitalized cases, and 110 samples from a healthcare worker study selected from a larger cohort of 1790 samples (20). These 110 samples were selected based on a high expected probability of concordance and discordance between the nucleocapsid IgG and RBD assays. For example, the sample was enriched for participants specifically who were nucleocapsid IgG^+^ and PCR^+^ (concordance) and for participants with COVID-19 symptoms and exposures who were nucleocapsid IgG^–^ (discordance). Thirty SARS-CoV-2^+^ samples were used from a study starting April 24, 2020, as comparators and two seropositive participants were resampled (16).

### Serological assay.

The ELISA protocol was previously described (16, 17). As in the prior assay, samples were run in duplicate and are reported as averages. Results were normalized to the CR3022 antibody with known affinity (Creative Biolabs, MRO-1214LC) (21). Participant sample anti-RBD IgG concentration (μg/ml) was calculated from the 4PL regression of the CR3022 calibration curve. A value of more than 0.39 μg/ml CR3022 was considered positive.

### Statistics.

Statistical analyses were performed with Prism (GraphPad) or R 4.2 (The R Foundation for Statistical Computing, http://www.R-project.org). The Wilcoxon-Mann-Whitney test was used to assess differences between groups; the Kruskal-Wallis test was used if more than 1 group was tested. Two-sample Kolmogorov-Smirnov test was used to compare essential versus nonessential samples. Changes over time were depicted with first order locally weighted regression and smoothing (Lowess model). An unpaired or paired 2-tailed *t* test was used to compare 2 groups, where appropriate. Pearson’s χ^2^ test statistic was used to compare proportions. *P* values of less than 0.05 were considered significant.

### Study approval.

All research activities were implemented under protocols approved by the IRB at Northwestern University (STU00206652, STU00212371, STU00212457, STU00212472, and STU00212515).

## Author contributions

LAV, NLR, MPV, EB, LMS, and RRH processed samples and/or performed ELISAs. ARD, LP, RS, and DTR managed data, analyzed data, and generated figures. TWM, EMM, BM, JTW, JFH, and MGI secured IRB approval and collected samples. ARD, BM, TWM, RTD, and EMM provided critical input into study design and wrote the manuscript. All authors reviewed and approved the final version of the manuscript.

## Supplementary Material

Trial reporting checklists

ICMJE disclosure forms

## Figures and Tables

**Table 1:**
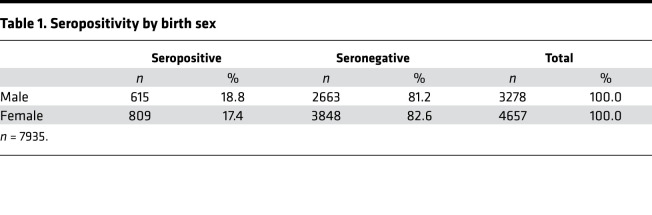
Seropositivity by birth sex

**Table 2:**
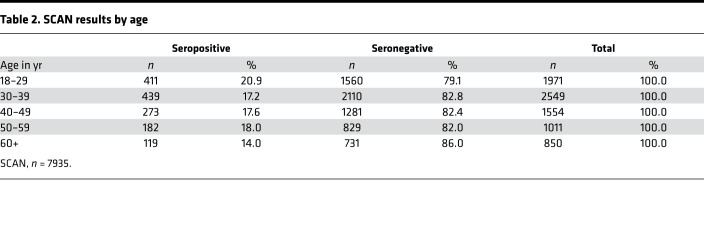
SCAN results by age

**Table 3:**
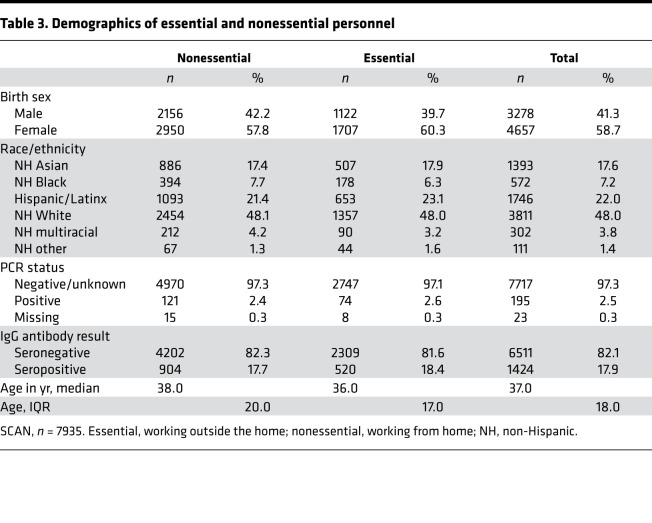
Demographics of essential and nonessential personnel

## Version 1 03/23/2021

In-Press Preview

## Version 2 05/10/2021

Electronic publication
