# Supplementary material for: Patterns and persistence of SARS-CoV-2 IgG antibodies in Chicago to monitor COVID-19 exposure
Source: JCI Insight. 2021 May 10;6(9):e146148. doi: 10.1172/jci.insight.146148 (PMC8262291; doi:10.1172/jci.insight.146148)
Supplement: ICMJE disclosure forms [file jciinsight-6-146148-s236.pdf]

ICMJE DISCLOSURE FORM

Date: 12 MAR 2021
Your Name: Alexis R Demonbreun
Manuscript Title: Patterns and persistence of SARS-CoV-2 IgG antibodies in Chicago to monitor COVID-19 exposure
Manuscript number (if known): 146148-INS-CMED-DN-2

In the interest of transparency, we ask you to disclose all relationships/activities/interests listed below that are related to the content of your manuscript. "Related" means any relation with for-profit or not-for-profit third parties whose interests may be affected by the content of the manuscript. Disclosure represents a commitment to transparency and does not necessarily indicate a bias. If you are in doubt about whether to list a relationship/activity/interest, it is preferable that you do so.

The following questions apply to the author's relationships/activities/interests as they relate to the current manuscript only.

The author's relationships/activities/interests should be defined broadly. For example, if your manuscript pertains to the epidemiology of hypertension, you should declare all relationships with manufacturers of antihypertensive medication, even if that medication is not mentioned in the manuscript.

In item #1 below, report all support for the work reported in this manuscript without time limit. For all other items, the time frame for disclosure is the past 36 months.

		**Name all entities with whom you have this relationship or indicate none (add rows as needed)**	**Specifications/Comments (e.g., if payments were made to you or to your institution)**
		**Time frame: Since the initial planning of the work**	
1	All support for the present manuscript (e.g., funding, provision of study materials, medical writing, article processing charges, etc.) **No time limit for this item.**	____None	Support from NSF, NIH, and generous donor gift as indicated in manuscript. All payments made to Northwestern.
		**Time frame: past 36 months**	
2	Grants or contracts from any entity (if not indicated in item #1 above).	_x__None	
3	Royalties or licenses	_x__None	
4	Consulting fees	_x__None	

5	Payment or honoraria for lectures, presentations, speakers bureaus, manuscript writing or educational events	x None	
6	Payment for expert testimony	x None	
7	Support for attending meetings and/or travel	x None	
8	Patents planned, issued or pending	x None	
9	Participation on a Data Safety Monitoring Board or Advisory Board	x None	
10	Leadership or fiduciary role in other board, society, committee or advocacy group, paid or unpaid	x None	
11	Stock or stock options	x None	
12	Receipt of equipment, materials, drugs, medical writing, gifts or other services	x None	
13	Other financial or non-financial interests	x None	

Please place an "X" next to the following statement to indicate your agreement:

x **I certify that I have answered every question and have not altered the wording of any of the questions on this form.**

ICMJE DISCLOSURE FORM

Date: 12 MAR 2021
Your Name: Thomas McDade
Manuscript Title: Patterns and persistence of SARS-CoV-2 IgG antibodies in Chicago to monitor COVID-19 exposure
Manuscript number (if known): 146148-INS-CMED-DN-2

In the interest of transparency, we ask you to disclose all relationships/activities/interests listed below that are related to the content of your manuscript. "Related" means any relation with for-profit or not-for-profit third parties whose interests may be affected by the content of the manuscript. Disclosure represents a commitment to transparency and does not necessarily indicate a bias. If you are in doubt about whether to list a relationship/activity/interest, it is preferable that you do so.

The following questions apply to the author's relationships/activities/interests as they relate to the current manuscript only.

The author's relationships/activities/interests should be defined broadly. For example, if your manuscript pertains to the epidemiology of hypertension, you should declare all relationships with manufacturers of antihypertensive medication, even if that medication is not mentioned in the manuscript.

In item #1 below, report all support for the work reported in this manuscript without time limit. For all other items, the time frame for disclosure is the past 36 months.

		Name all entities with whom you have this relationship or indicate none (add rows as needed)	Specifications/Comments (e.g., if payments were made to you or to your institution)
		**Time frame: Since the initial planning of the work**	
1	All support for the present manuscript (e.g., funding, provision of study materials, medical writing, article processing charges, etc.) **No time limit for this item.**	____None	Support from NSF, NIH, and generous donor gift as indicated in manuscript. All payments made to Northwestern.
		**Time frame: past 36 months**	
2	Grants or contracts from any entity (if not indicated in item #1 above).	_x__None	
3	Royalties or licenses	_x__None	
4	Consulting fees	_x__None	

5	Payment or honoraria for lectures, presentations, speakers bureaus, manuscript writing or educational events	__x__None	
6	Payment for expert testimony	__x__None	
7	Support for attending meetings and/or travel	__x__None	
8	Patents planned, issued or pending	__x__None	
9	Participation on a Data Safety Monitoring Board or Advisory Board	__x__None	
10	Leadership or fiduciary role in other board, society, committee or advocacy group, paid or unpaid	__x__None	
11	Stock or stock options	__x__None	
12	Receipt of equipment, materials, drugs, medical writing, gifts or other services	__x__None	
13	Other financial or non-financial interests	x	Financial interest in EnMed Microanalytics, a laboratory testing company.

Please place an "X" next to the following statement to indicate your agreement:

x___ I certify that I have answered every question and have not altered the wording of any of the questions on this form.

ICMJE DISCLOSURE FORM

Date: 12 MAR 2021
Your Name: Lorenzo Pesce
Manuscript Title: Patterns and persistence of SARS-CoV-2 IgG antibodies in Chicago to monitor COVID-19 exposure
Manuscript number (if known): 146148-INS-CMED-DN-2

In the interest of transparency, we ask you to disclose all relationships/activities/interests listed below that are related to the content of your manuscript. "Related" means any relation with for-profit or not-for-profit third parties whose interests may be affected by the content of the manuscript. Disclosure represents a commitment to transparency and does not necessarily indicate a bias. If you are in doubt about whether to list a relationship/activity/interest, it is preferable that you do so.

The following questions apply to the author's relationships/activities/interests as they relate to the current manuscript only.

The author's relationships/activities/interests should be defined broadly. For example, if your manuscript pertains to the epidemiology of hypertension, you should declare all relationships with manufacturers of antihypertensive medication, even if that medication is not mentioned in the manuscript.

In item #1 below, report all support for the work reported in this manuscript without time limit. For all other items, the time frame for disclosure is the past 36 months.

		Name all entities with whom you have this relationship or indicate none (add rows as needed)	Specifications/Comments (e.g., if payments were made to you or to your institution)
**Time frame: Since the initial planning of the work**			
1	All support for the present manuscript (e.g., funding, provision of study materials, medical writing, article processing charges, etc.) **No time limit for this item.**	____None	Support from NSF, NIH, and generous donor gift as indicated in manuscript. All payments made to Northwestern.
**Time frame: past 36 months**			
2	Grants or contracts from any entity (if not indicated in item #1 above).	x None	
3	Royalties or licenses	x None	
4	Consulting fees	x None	

5	Payment or honoraria for lectures, presentations, speakers bureaus, manuscript writing or educational events	__x__None	
6	Payment for expert testimony	__x__None	
7	Support for attending meetings and/or travel	__x__None	
8	Patents planned, issued or pending	__x__None	
9	Participation on a Data Safety Monitoring Board or Advisory Board	__x__None	
10	Leadership or fiduciary role in other board, society, committee or advocacy group, paid or unpaid	__x__None	
11	Stock or stock options	__x__None	
12	Receipt of equipment, materials, drugs, medical writing, gifts or other services	__x__None	
13	Other financial or non-financial interests	__x__None	

Please place an "X" next to the following statement to indicate your agreement:

x___ **I certify that I have answered every question and have not altered the wording of any of the questions on this form.**

ICMJE DISCLOSURE FORM

Date: 12 MAR 2021
Your Name: Nina L. Reiser
Manuscript Title: Patterns and persistence of SARS-CoV-2 IgG antibodies in Chicago to monitor COVID-19 exposure
Manuscript number (if known): 146148-INS-CMED-DN-2

In the interest of transparency, we ask you to disclose all relationships/activities/interests listed below that are related to the content of your manuscript. "Related" means any relation with for-profit or not-for-profit third parties whose interests may be affected by the content of the manuscript. Disclosure represents a commitment to transparency and does not necessarily indicate a bias. If you are in doubt about whether to list a relationship/activity/interest, it is preferable that you do so.

The following questions apply to the author's relationships/activities/interests as they relate to the current manuscript only.

The author's relationships/activities/interests should be defined broadly. For example, if your manuscript pertains to the epidemiology of hypertension, you should declare all relationships with manufacturers of antihypertensive medication, even if that medication is not mentioned in the manuscript.

In item #1 below, report all support for the work reported in this manuscript without time limit. For all other items, the time frame for disclosure is the past 36 months.

		Name all entities with whom you have this relationship or indicate none (add rows as needed)	Specifications/Comments (e.g., if payments were made to you or to your institution)
**Time frame: Since the initial planning of the work**			
1	All support for the present manuscript (e.g., funding, provision of study materials, medical writing, article processing charges, etc.) **No time limit for this item.**	____None	Support from NSF, NIH, and generous donor gift as indicated in manuscript. All payments made to Northwestern.
**Time frame: past 36 months**			
2	Grants or contracts from any entity (if not indicated in item #1 above).	x None	
3	Royalties or licenses	x None	
4	Consulting fees	x None	

5	Payment or honoraria for lectures, presentations, speakers bureaus, manuscript writing or educational events	_x__None	
6	Payment for expert testimony	_x__None	
7	Support for attending meetings and/or travel	_x__None	
8	Patents planned, issued or pending	_x__None	
9	Participation on a Data Safety Monitoring Board or Advisory Board	_x__None	
10	Leadership or fiduciary role in other board, society, committee or advocacy group, paid or unpaid	_x__None	
11	Stock or stock options	_x__None	
12	Receipt of equipment, materials, drugs, medical writing, gifts or other services	_x__None	
13	Other financial or non-financial interests	_x__None	

Please place an "X" next to the following statement to indicate your agreement:

x___ I certify that I have answered every question and have not altered the wording of any of the questions on this form.

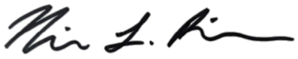

ICMJE DISCLOSURE FORM

Date: 12 MAR 2021

Your Name: Nina L. Reiser

Manuscript Title: Patterns and persistence of SARS-CoV-2 IgG antibodies in Chicago to monitor COVID-19 exposure

Manuscript number (if known): 146148-INS-CMED-DN-2

In the interest of transparency, we ask you to disclose all relationships/activities/interests listed below that are related to the content of your manuscript. "Related" means any relation with for-profit or not-for-profit third parties whose interests may be affected by the content of the manuscript. Disclosure represents a commitment to transparency and does not necessarily indicate a bias. If you are in doubt about whether to list a relationship/activity/interest, it is preferable that you do so.

The following questions apply to the author's relationships/activities/interests as they relate to the current manuscript only.

The author's relationships/activities/interests should be defined broadly. For example, if your manuscript pertains to the epidemiology of hypertension, you should declare all relationships with manufacturers of antihypertensive medication, even if that medication is not mentioned in the manuscript.

In item #1 below, report all support for the work reported in this manuscript without time limit. For all other items, the time frame for disclosure is the past 36 months.

		Name all entities with whom you have this relationship or indicate none (add rows as needed)	Specifications/Comments (e.g., if payments were made to you or to your institution)
		**Time frame: Since the initial planning of the work**	
1	All support for the present manuscript (e.g., funding, provision of study materials, medical writing, article processing charges, etc.) **No time limit for this item.**	____None	Support from NSF, NIH, and generous donor gift as indicated in manuscript. All payments made to Northwestern.
		**Time frame: past 36 months**	
2	Grants or contracts from any entity (if not indicated in item #1 above).	x None	
3	Royalties or licenses	x None	
4	Consulting fees	x None	

5	Payment or honoraria for lectures, presentations, speakers bureaus, manuscript writing or educational events	__x__None	
6	Payment for expert testimony	__x__None	
7	Support for attending meetings and/or travel	__x__None	
8	Patents planned, issued or pending	__x__None	
9	Participation on a Data Safety Monitoring Board or Advisory Board	__x__None	
10	Leadership or fiduciary role in other board, society, committee or advocacy group, paid or unpaid	__x__None	
11	Stock or stock options	__x__None	
12	Receipt of equipment, materials, drugs, medical writing, gifts or other services	__x__None	
13	Other financial or non-financial interests	__x__None	

Please place an "X" next to the following statement to indicate your agreement:

_x___ I certify that I have answered every question and have not altered the wording of any of the questions on this form.

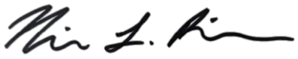

ICMJE DISCLOSURE FORM

Date: 12 MAR 2021
Your Name: Elena Bogdanovic
Manuscript Title: Patterns and persistence of SARS-CoV-2 IgG antibodies in Chicago to monitor COVID-19 exposure
Manuscript number (if known): 146148-INS-CMED-DN-2

In the interest of transparency, we ask you to disclose all relationships/activities/interests listed below that are related to the content of your manuscript. "Related" means any relation with for-profit or not-for-profit third parties whose interests may be affected by the content of the manuscript. Disclosure represents a commitment to transparency and does not necessarily indicate a bias. If you are in doubt about whether to list a relationship/activity/interest, it is preferable that you do so.

The following questions apply to the author's relationships/activities/interests as they relate to the current manuscript only.

The author's relationships/activities/interests should be defined broadly. For example, if your manuscript pertains to the epidemiology of hypertension, you should declare all relationships with manufacturers of antihypertensive medication, even if that medication is not mentioned in the manuscript.

In item #1 below, report all support for the work reported in this manuscript without time limit. For all other items, the time frame for disclosure is the past 36 months.

		Name all entities with whom you have this relationship or indicate none (add rows as needed)	Specifications/Comments (e.g., if payments were made to you or to your institution)
		Time frame: Since the initial planning of the work	
1	All support for the present manuscript (e.g., funding, provision of study materials, medical writing, article processing charges, etc.) **No time limit for this item.**	____None	Support from NSF, NIH, and generous donor gift as indicated in manuscript. All payments made to Northwestern.
		Time frame: past 36 months	
2	Grants or contracts from any entity (if not indicated in item #1 above).	x None	
3	Royalties or licenses	x None	
4	Consulting fees	x None	

5	Payment or honoraria for lectures, presentations, speakers bureaus, manuscript writing or educational events	_x_ None	
6	Payment for expert testimony	_x_ None	
7	Support for attending meetings and/or travel	_x_ None	
8	Patents planned, issued or pending	_x_ None	
9	Participation on a Data Safety Monitoring Board or Advisory Board	_x_ None	
10	Leadership or fiduciary role in other board, society, committee or advocacy group, paid or unpaid	_x_ None	
11	Stock or stock options	_x_ None	
12	Receipt of equipment, materials, drugs, medical writing, gifts or other services	_x_ None	
13	Other financial or non-financial interests	_x_ None	

Please place an "X" next to the following statement to indicate your agreement:

EBogdani

x___ I certify that I have answered every question and have not altered the wording of any of the questions on this form.

ICMJE DISCLOSURE FORM

Date: 12 MAR 2021
Your Name: Matthew P Velez
Manuscript Title: Patterns and persistence of SARS-CoV-2 IgG antibodies in Chicago to monitor COVID-19 exposure
Manuscript number (if known): 146148-INS-CMED-DN-2

In the interest of transparency, we ask you to disclose all relationships/activities/interests listed below that are related to the content of your manuscript. "Related" means any relation with for-profit or not-for-profit third parties whose interests may be affected by the content of the manuscript. Disclosure represents a commitment to transparency and does not necessarily indicate a bias. If you are in doubt about whether to list a relationship/activity/interest, it is preferable that you do so.

The following questions apply to the author's relationships/activities/interests as they relate to the current manuscript only.

The author's relationships/activities/interests should be defined broadly. For example, if your manuscript pertains to the epidemiology of hypertension, you should declare all relationships with manufacturers of antihypertensive medication, even if that medication is not mentioned in the manuscript.

In item #1 below, report all support for the work reported in this manuscript without time limit. For all other items, the time frame for disclosure is the past 36 months.

		Name all entities with whom you have this relationship or indicate none (add rows as needed)	Specifications/Comments (e.g., if payments were made to you or to your institution)
**Time frame: Since the initial planning of the work**			
1	All support for the present manuscript (e.g., funding, provision of study materials, medical writing, article processing charges, etc.) **No time limit for this item.**	____None	Support from NSF, NIH, and generous donor gift as indicated
**Time frame: past 36 months**			
2	Grants or contracts from any entity (if not indicated in item #1 above).	x None	
3	Royalties or licenses	x None	
4	Consulting fees	x None	

5	Payment or honoraria for lectures, presentations, speakers bureaus, manuscript writing or educational events	x None	
6	Payment for expert testimony	x None	
7	Support for attending meetings and/or travel	x None	
8	Patents planned, issued or pending	x None	
9	Participation on a Data Safety Monitoring Board or Advisory Board	x None	
10	Leadership or fiduciary role in other board, society, committee or advocacy group, paid or unpaid	x None	
11	Stock or stock options	x None	
12	Receipt of equipment, materials, drugs, medical writing, gifts or other services	x None	
13	Other financial or non-financial interests	x None	

Please place an "X" next to the following statement to indicate your agreement:

x **I certify that I have answered every question and have not altered the wording of any of the questions on this form.**

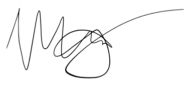

ICMJE DISCLOSURE FORM

Date: 12 MAR 2021
Your Name: Ryan Hsieh
Manuscript Title: Patterns and persistence of SARS-CoV-2 IgG antibodies in Chicago to monitor COVID-19 exposure
Manuscript number (if known): 146148-INS-CMED-DN-2

In the interest of transparency, we ask you to disclose all relationships/activities/interests listed below that are related to the content of your manuscript. "Related" means any relation with for-profit or not-for-profit third parties whose interests may be affected by the content of the manuscript. Disclosure represents a commitment to transparency and does not necessarily indicate a bias. If you are in doubt about whether to list a relationship/activity/interest, it is preferable that you do so.

The following questions apply to the author's relationships/activities/interests as they relate to the current manuscript only.

The author's relationships/activities/interests should be defined broadly. For example, if your manuscript pertains to the epidemiology of hypertension, you should declare all relationships with manufacturers of antihypertensive medication, even if that medication is not mentioned in the manuscript.

In item #1 below, report all support for the work reported in this manuscript without time limit. For all other items, the time frame for disclosure is the past 36 months.

		Name all entities with whom you have this relationship or indicate none (add rows as needed)	Specifications/Comments (e.g., if payments were made to you or to your institution)
**Time frame: Since the initial planning of the work**			
1	All support for the present manuscript (e.g., funding, provision of study materials, medical writing, article processing charges, etc.) **No time limit for this item.**	____None	Support from NSF, NIH, and generous donor gift as indicated in manuscript. All payments made to Northwestern.
**Time frame: past 36 months**			
2	Grants or contracts from any entity (if not indicated in item #1 above).	_x_None	
3	Royalties or licenses	_x_None	
4	Consulting fees	_x_None	

5	Payment or honoraria for lectures, presentations, speakers bureaus, manuscript writing or educational events	__x__None	
6	Payment for expert testimony	__x__None	
7	Support for attending meetings and/or travel	__x__None	
8	Patents planned, issued or pending	__x__None	
9	Participation on a Data Safety Monitoring Board or Advisory Board	__x__None	
10	Leadership or fiduciary role in other board, society, committee or advocacy group, paid or unpaid	__x__None	
11	Stock or stock options	__x__None	
12	Receipt of equipment, materials, drugs, medical writing, gifts or other services	__x__None	
13	Other financial or non-financial interests	__x__None	

Please place an "X" next to the following statement to indicate your agreement:

x___ **I certify that I have answered every question and have not altered the wording of any of the questions on this form.**

Ryan Hsieh

ICMJE DISCLOSURE FORM

Date: 12 MAR 2021
Your Name: Lacy Simons
Manuscript Title: Patterns and persistence of SARS-CoV-2 IgG antibodies in Chicago to monitor COVID-19 exposure
Manuscript number (if known): 146148-INS-CMED-DN-2

In the interest of transparency, we ask you to disclose all relationships/activities/interests listed below that are related to the content of your manuscript. "Related" means any relation with for-profit or not-for-profit third parties whose interests may be affected by the content of the manuscript. Disclosure represents a commitment to transparency and does not necessarily indicate a bias. If you are in doubt about whether to list a relationship/activity/interest, it is preferable that you do so.

The following questions apply to the author's relationships/activities/interests as they relate to the current manuscript only.

The author's relationships/activities/interests should be defined broadly. For example, if your manuscript pertains to the epidemiology of hypertension, you should declare all relationships with manufacturers of antihypertensive medication, even if that medication is not mentioned in the manuscript.

In item #1 below, report all support for the work reported in this manuscript without time limit. For all other items, the time frame for disclosure is the past 36 months.

		Name all entities with whom you have this relationship or indicate none (add rows as needed)	Specifications/Comments (e.g., if payments were made to you or to your institution)
**Time frame: Since the initial planning of the work**			
1	All support for the present manuscript (e.g., funding, provision of study materials, medical writing, article processing charges, etc.) **No time limit for this item.**	____None	Support from NSF, NIH, and generous donor gift as indicated in manuscript. All payments made to Northwestern.
**Time frame: past 36 months**			
2	Grants or contracts from any entity (if not indicated in item #1 above).	_x__None	
3	Royalties or licenses	_x__None	
4	Consulting fees	_x__None	

5	Payment or honoraria for lectures, presentations, speakers bureaus, manuscript writing or educational events	x None	
6	Payment for expert testimony	x None	
7	Support for attending meetings and/or travel	x None	
8	Patents planned, issued or pending	x None	
9	Participation on a Data Safety Monitoring Board or Advisory Board	x None	
10	Leadership or fiduciary role in other board, society, committee or advocacy group, paid or unpaid	x None	
11	Stock or stock options	x None	
12	Receipt of equipment, materials, drugs, medical writing, gifts or other services	x None	
13	Other financial or non-financial interests	x None	

Please place an "X" next to the following statement to indicate your agreement:

x **I certify that I have answered every question and have not altered the wording of any of the questions on this form.**

Lacy Simons

ICMJE DISCLOSURE FORM

Date: 12 MAR 2021
Your Name: Rana Saber
Manuscript Title: Patterns and persistence of SARS-CoV-2 IgG antibodies in Chicago to monitor COVID-19 exposure
Manuscript number (if known): 146148-INS-CMED-DN-2

In the interest of transparency, we ask you to disclose all relationships/activities/interests listed below that are related to the content of your manuscript. "Related" means any relation with for-profit or not-for-profit third parties whose interests may be affected by the content of the manuscript. Disclosure represents a commitment to transparency and does not necessarily indicate a bias. If you are in doubt about whether to list a relationship/activity/interest, it is preferable that you do so.

The following questions apply to the author's relationships/activities/interests as they relate to the current manuscript only.

The author's relationships/activities/interests should be defined broadly. For example, if your manuscript pertains to the epidemiology of hypertension, you should declare all relationships with manufacturers of antihypertensive medication, even if that medication is not mentioned in the manuscript.

In item #1 below, report all support for the work reported in this manuscript without time limit. For all other items, the time frame for disclosure is the past 36 months.

		**Name all entities with whom you have this relationship or indicate none (add rows as needed)**	**Specifications/Comments (e.g., if payments were made to you or to your institution)**
		**Time frame: Since the initial planning of the work**	
1	All support for the present manuscript (e.g., funding, provision of study materials, medical writing, article processing charges, etc.) **No time limit for this item.**	____None	Support from NSF, NIH, and generous donor gift as indicated in manuscript. All payments made to Northwestern.
		**Time frame: past 36 months**	
2	Grants or contracts from any entity (if not indicated in item #1 above).	x None	
3	Royalties or licenses	x None	
4	Consulting fees	x None	

5	Payment or honoraria for lectures, presentations, speakers bureaus, manuscript writing or educational events	x None	
6	Payment for expert testimony	x None	
7	Support for attending meetings and/or travel	x None	
8	Patents planned, issued or pending	x None	
9	Participation on a Data Safety Monitoring Board or Advisory Board	x None	
10	Leadership or fiduciary role in other board, society, committee or advocacy group, paid or unpaid	x None	
11	Stock or stock options	x None	
12	Receipt of equipment, materials, drugs, medical writing, gifts or other services	x None	
13	Other financial or non-financial interests	x None	

Please place an "X" next to the following statement to indicate your agreement:

x **I certify that I have answered every question and have not altered the wording of any of the questions on this form.**

Rana Saber

ICMJE DISCLOSURE FORM

Date: 12 MAR 2021
Your Name: Daniel T Ryan
Manuscript Title: Patterns and persistence of SARS-CoV-2 IgG antibodies in Chicago to monitor COVID-19 exposure
Manuscript number (if known): 146148-INS-CMED-DN-2

In the interest of transparency, we ask you to disclose all relationships/activities/interests listed below that are related to the content of your manuscript. "Related" means any relation with for-profit or not-for-profit third parties whose interests may be affected by the content of the manuscript. Disclosure represents a commitment to transparency and does not necessarily indicate a bias. If you are in doubt about whether to list a relationship/activity/interest, it is preferable that you do so.

The following questions apply to the author's relationships/activities/interests as they relate to the current manuscript only.

The author's relationships/activities/interests should be defined broadly. For example, if your manuscript pertains to the epidemiology of hypertension, you should declare all relationships with manufacturers of antihypertensive medication, even if that medication is not mentioned in the manuscript.

In item #1 below, report all support for the work reported in this manuscript without time limit. For all other items, the time frame for disclosure is the past 36 months.

		Name all entities with whom you have this relationship or indicate none (add rows as needed)	Specifications/Comments (e.g., if payments were made to you or to your institution)
		**Time frame: Since the initial planning of the work**	
1	All support for the present manuscript (e.g., funding, provision of study materials, medical writing, article processing charges, etc.) **No time limit for this item.**	____None	Support from NSF, NIH, and generous donor gift as indicated in manuscript. All payments made to Northwestern.
		**Time frame: past 36 months**	
2	Grants or contracts from any entity (if not indicated in item #1 above).	_x__None	
3	Royalties or licenses	_x__None	
4	Consulting fees	_x__None	

5	Payment or honoraria for lectures, presentations, speakers bureaus, manuscript writing or educational events	_x_None	
6	Payment for expert testimony	_x_None	
7	Support for attending meetings and/or travel	_x_None	
8	Patents planned, issued or pending	_x_None	
9	Participation on a Data Safety Monitoring Board or Advisory Board	_x_None	
10	Leadership or fiduciary role in other board, society, committee or advocacy group, paid or unpaid	_x_None	
11	Stock or stock options	_x_None	
12	Receipt of equipment, materials, drugs, medical writing, gifts or other services	_x_None	
13	Other financial or non-financial interests	_x_None	

Please place an "X" next to the following statement to indicate your agreement:

_x_ **I certify that I have answered every question and have not altered the wording of any of the questions on this form.**

ICMJE DISCLOSURE FORM

Date:__13 March 2021____
Your Name:_Michael G. Ison____
Manuscript Title: Patterns and persistence of SARS-CoV-2 IgG antibodies in Chicago to monitor COVID-19 exposure
Manuscript number (if known):_ 146148-INS-CMED-DN-2

In the interest of transparency, we ask you to disclose all relationships/activities/interests listed below that are related to the content of your manuscript. "Related" means any relation with for-profit or not-for-profit third parties whose interests may be affected by the content of the manuscript. Disclosure represents a commitment to transparency and does not necessarily indicate a bias. If you are in doubt about whether to list a relationship/activity/interest, it is preferable that you do so.

The following questions apply to the author's relationships/activities/interests as they relate to the current manuscript only.

The author's relationships/activities/interests should be defined broadly. For example, if your manuscript pertains to the epidemiology of hypertension, you should declare all relationships with manufacturers of antihypertensive medication, even if that medication is not mentioned in the manuscript.

In item #1 below, report all support for the work reported in this manuscript without time limit. For all other items, the time frame for disclosure is the past 36 months.

		**Name all entities with whom you have this relationship or indicate none (add rows as needed)**	**Specifications/Comments (e.g., if payments were made to you or to your institution)**
		**Time frame: Since the initial planning of the work**	
1	All support for the present manuscript (e.g., funding, provision of study materials, medical writing, article processing charges, etc.) **No time limit for this item.**	__X__None	
		**Time frame: past 36 months**	
2	Grants or contracts from any entity (if not indicated in item #1 above).	____None	AiCuris, Shire
3	Royalties or licenses	____None	UpToDate

4	Consulting fees	____None	Adagio, AlloVir, Celltrion, Cidara, Genentech/Roche, Janssen, Shionogi, Takeda, Viracor Eurofin
5	Payment or honoraria for lectures, presentations, speakers bureaus, manuscript writing or educational events	__X__None	
6	Payment for expert testimony	__X__None	
7	Support for attending meetings and/or travel	__X__None	
8	Patents planned, issued or pending	__X__None	
9	Participation on a Data Safety Monitoring Board or Advisory Board	____None	NIH, Janssen, Merck, SAB Biotherapeutics, Sequiris, Takeda, Vitaeris
10	Leadership or fiduciary role in other board, society, committee or advocacy group, paid or unpaid	__X__None	
11	Stock or stock options	__X__None	
12	Receipt of equipment, materials, drugs, medical writing, gifts or other services	__X__None	
13	Other financial or non-financial interests	__X__None	

Please place an "X" next to the following statement to indicate your agreement:

__X_ I certify that I have answered every question and have not altered the wording of any of the questions on this form.

ICMJE DISCLOSURE FORM

Date: 12 MAR 2021
Your Name: Judd Hultquist
Manuscript Title: Patterns and persistence of SARS-CoV-2 IgG antibodies in Chicago to monitor COVID-19 exposure
Manuscript number (if known): 146148-INS-CMED-DN-2

In the interest of transparency, we ask you to disclose all relationships/activities/interests listed below that are related to the content of your manuscript. "Related" means any relation with for-profit or not-for-profit third parties whose interests may be affected by the content of the manuscript. Disclosure represents a commitment to transparency and does not necessarily indicate a bias. If you are in doubt about whether to list a relationship/activity/interest, it is preferable that you do so.

The following questions apply to the author's relationships/activities/interests as they relate to the current manuscript only.

The author's relationships/activities/interests should be defined broadly. For example, if your manuscript pertains to the epidemiology of hypertension, you should declare all relationships with manufacturers of antihypertensive medication, even if that medication is not mentioned in the manuscript.

In item #1 below, report all support for the work reported in this manuscript without time limit. For all other items, the time frame for disclosure is the past 36 months.

		Name all entities with whom you have this relationship or indicate none (add rows as needed)	Specifications/Comments (e.g., if payments were made to you or to your institution)
**Time frame: Since the initial planning of the work**			
1	All support for the present manuscript (e.g., funding, provision of study materials, medical writing, article processing charges, etc.) **No time limit for this item.**	____None	Support from NSF, NIH, and generous donor gift as indicated in manuscript. All payments made to Northwestern.
**Time frame: past 36 months**			
2	Grants or contracts from any entity (if not indicated in item #1 above).	__x__None	
3	Royalties or licenses	__x__None	
4	Consulting fees	__x__None	

5	Payment or honoraria for lectures, presentations, speakers bureaus, manuscript writing or educational events	x None	
6	Payment for expert testimony	x None	
7	Support for attending meetings and/or travel	x None	
8	Patents planned, issued or pending	x None	
9	Participation on a Data Safety Monitoring Board or Advisory Board	x None	
10	Leadership or fiduciary role in other board, society, committee or advocacy group, paid or unpaid	x None	
11	Stock or stock options	x None	
12	Receipt of equipment, materials, drugs, medical writing, gifts or other services	x None	
13	Other financial or non-financial interests	x None	

Please place an "X" next to the following statement to indicate your agreement:

x **I certify that I have answered every question and have not altered the wording of any of the questions on this form.**

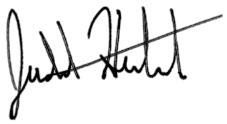

ICMJE DISCLOSURE FORM

Date: 12 MAR 2021
Your Name: John T. Wilkins, MD, MS
Manuscript Title: Patterns and persistence of SARS-CoV-2 IgG antibodies in Chicago to monitor COVID-19 exposure
Manuscript number (if known): 146148-INS-CMED-DN-2

In the interest of transparency, we ask you to disclose all relationships/activities/interests listed below that are related to the content of your manuscript. "Related" means any relation with for-profit or not-for-profit third parties whose interests may be affected by the content of the manuscript. Disclosure represents a commitment to transparency and does not necessarily indicate a bias. If you are in doubt about whether to list a relationship/activity/interest, it is preferable that you do so.

The following questions apply to the author's relationships/activities/interests as they relate to the current manuscript only.

The author's relationships/activities/interests should be defined broadly. For example, if your manuscript pertains to the epidemiology of hypertension, you should declare all relationships with manufacturers of antihypertensive medication, even if that medication is not mentioned in the manuscript.

In item #1 below, report all support for the work reported in this manuscript without time limit. For all other items, the time frame for disclosure is the past 36 months.

		**Name all entities with whom you have this relationship or indicate none (add rows as needed)**	**Specifications/Comments (e.g., if payments were made to you or to your institution)**
		**Time frame: Since the initial planning of the work**	
1	All support for the present manuscript (e.g., funding, provision of study materials, medical writing, article processing charges, etc.) **No time limit for this item.**	____None	Support from NSF, NIH, and generous donor gift as indicated in manuscript. All payments made to Northwestern.
		**Time frame: past 36 months**	
2	Grants or contracts from any entity (if not indicated in item #1 above).	_x__None	
3	Royalties or licenses	_x__None	
4	Consulting fees	_x__None	

5	Payment or honoraria for lectures, presentations, speakers bureaus, manuscript writing or educational events	__x__None	
6	Payment for expert testimony	__x__None	
7	Support for attending meetings and/or travel	__x__None	
8	Patents planned, issued or pending	__x__None	
9	Participation on a Data Safety Monitoring Board or Advisory Board	__x__None	
10	Leadership or fiduciary role in other board, society, committee or advocacy group, paid or unpaid	__x__None	
11	Stock or stock options	__x__None	
12	Receipt of equipment, materials, drugs, medical writing, gifts or other services	__x__None	
13	Other financial or non-financial interests	__x__None	

Please place an "X" next to the following statement to indicate your agreement:

x___ **I certify that I have answered every question and have not altered the wording of any of the questions on this form.**

ICMJE DISCLOSURE FORM

Date: 12 MAR 2021
Your Name: Richard T. D'Aquila
Manuscript Title: Patterns and persistence of SARS-CoV-2 IgG antibodies in Chicago to monitor COVID-19 exposure
Manuscript number (if known): 146148-INS-CMED-DN-2

In the interest of transparency, we ask you to disclose all relationships/activities/interests listed below that are related to the content of your manuscript. “Related” means any relation with for-profit or not-for-profit third parties whose interests may be affected by the content of the manuscript. Disclosure represents a commitment to transparency and does not necessarily indicate a bias. If you are in doubt about whether to list a relationship/activity/interest, it is preferable that you do so.

The following questions apply to the author’s relationships/activities/interests as they relate to the current manuscript only.

The author’s relationships/activities/interests should be defined broadly. For example, if your manuscript pertains to the epidemiology of hypertension, you should declare all relationships with manufacturers of antihypertensive medication, even if that medication is not mentioned in the manuscript.

In item #1 below, report all support for the work reported in this manuscript without time limit. For all other items, the time frame for disclosure is the past 36 months.

		Name all entities with whom you have this relationship or indicate none (add rows as needed)	Specifications/Comments (e.g., if payments were made to you or to your institution)
**Time frame: Since the initial planning of the work**			
1	All support for the present manuscript (e.g., funding, provision of study materials, medical writing, article processing charges, etc.) **No time limit for this item.**	____None	Support from NSF, NIH, and generous donor gift as indicated in manuscript. All payments made to Northwestern.
**Time frame: past 36 months**			
2	Grants or contracts from any entity (if not indicated in item #1 above).	x None	
3	Royalties or licenses	x None	
4	Consulting fees	x None	

5	Payment or honoraria for lectures, presentations, speakers bureaus, manuscript writing or educational events	__x__None	
6	Payment for expert testimony	__x__None	
7	Support for attending meetings and/or travel	__x__None	
8	Patents planned, issued or pending	US patent 9,688,637	3-Aminobenzamides and uses thereof for increasing cellular levels of APOBEC3G (A3G) and other A3 family members. issued June 27, 2017. No payments
9	Participation on a Data Safety Monitoring Board or Advisory Board	__x__None	
10	Leadership or fiduciary role in other board, society, committee or advocacy group, paid or unpaid	__x__None	
11	Stock or stock options	__x__None	
12	Receipt of equipment, materials, drugs, medical writing, gifts or other services	__x__None	
13	Other financial or non-financial interests	__x__None	

Please place an "X" next to the following statement to indicate your agreement:

x___ I certify that I have answered every question and have not altered the wording of any of the questions on this form.

ICMJE DISCLOSURE FORM

Date: 12 MAR 2021

Your Name: Brian Mustanski

Manuscript Title: Patterns and persistence of SARS-CoV-2 IgG antibodies in Chicago to monitor COVID-19 exposure

Manuscript number (if known): 146148-INS-CMED-DN-2

In the interest of transparency, we ask you to disclose all relationships/activities/interests listed below that are related to the content of your manuscript. "Related" means any relation with for-profit or not-for-profit third parties whose interests may be affected by the content of the manuscript. Disclosure represents a commitment to transparency and does not necessarily indicate a bias. If you are in doubt about whether to list a relationship/activity/interest, it is preferable that you do so.

The following questions apply to the author's relationships/activities/interests as they relate to the current manuscript only.

The author's relationships/activities/interests should be defined broadly. For example, if your manuscript pertains to the epidemiology of hypertension, you should declare all relationships with manufacturers of antihypertensive medication, even if that medication is not mentioned in the manuscript.

In item #1 below, report all support for the work reported in this manuscript without time limit. For all other items, the time frame for disclosure is the past 36 months.

		**Name all entities with whom you have this relationship or indicate none (add rows as needed)**	**Specifications/Comments (e.g., if payments were made to you or to your institution)**
		**Time frame: Since the initial planning of the work**	
1	All support for the present manuscript (e.g., funding, provision of study materials, medical writing, article processing charges, etc.) **No time limit for this item.**	____None	Support from NSF, NIH, and generous donor gift as indicated in manuscript. All payments made to Northwestern.
		**Time frame: past 36 months**	
2	Grants or contracts from any entity (if not indicated in item #1 above).	_x__None	
3	Royalties or licenses	_x__None	
4	Consulting fees	_x__None	

5	Payment or honoraria for lectures, presentations, speakers bureaus, manuscript writing or educational events	__x__None	
6	Payment for expert testimony	__x__None	
7	Support for attending meetings and/or travel	__x__None	
8	Patents planned, issued or pending	__x__None	
9	Participation on a Data Safety Monitoring Board or Advisory Board	__x__None	
10	Leadership or fiduciary role in other board, society, committee or advocacy group, paid or unpaid	__x__None	
11	Stock or stock options	__x__None	
12	Receipt of equipment, materials, drugs, medical writing, gifts or other services	__x__None	
13	Other financial or non-financial interests	__x__None	

Please place an "X" next to the following statement to indicate your agreement:

x___ **I certify that I have answered every question and have not altered the wording of any of the questions on this form.**

ICMJE DISCLOSURE FORM

Date:______12 MAR 2021______

Your Name:______________________

Manuscript Title:__Patterns and persistence of SARS-CoV-2 IgG antibodies in Chicago to monitor COVID-19 exposure__

Manuscript number (if known):__146148-INS-CMED-DN-2__

In the interest of transparency, we ask you to disclose all relationships/activities/interests listed below that are related to the content of your manuscript. "Related" means any relation with for-profit or not-for-profit third parties whose interests may be affected by the content of the manuscript. Disclosure represents a commitment to transparency and does not necessarily indicate a bias. If you are in doubt about whether to list a relationship/activity/interest, it is preferable that you do so.

The following questions apply to the author's relationships/activities/interests as they relate to the current manuscript only.

The author's relationships/activities/interests should be defined broadly. For example, if your manuscript pertains to the epidemiology of hypertension, you should declare all relationships with manufacturers of antihypertensive medication, even if that medication is not mentioned in the manuscript.

In item #1 below, report all support for the work reported in this manuscript without time limit. For all other items, the time frame for disclosure is the past 36 months.

		**Name all entities with whom you have this relationship or indicate none (add rows as needed)**	**Specifications/Comments (e.g., if payments were made to you or to your institution)**
		**Time frame: Since the initial planning of the work**	
1	All support for the present manuscript (e.g., funding, provision of study materials, medical writing, article processing charges, etc.) **No time limit for this item.**	____None	Support from NSF, NIH, and generous donor gift as indicated in manuscript. All payments made to Northwestern.
		**Time frame: past 36 months**	
2	Grants or contracts from any entity (if not indicated in item #1 above).	_x__None	
3	Royalties or licenses	_x__None	
4	Consulting fees	_x__None	

5	Payment or honoraria for lectures, presentations, speakers bureaus, manuscript writing or educational events	__x__None	
6	Payment for expert testimony	__x__None	
7	Support for attending meetings and/or travel	__x__None	
8	Patents planned, issued or pending	__x__None	
9	Participation on a Data Safety Monitoring Board or Advisory Board	__x__None	
10	Leadership or fiduciary role in other board, society, committee or advocacy group, paid or unpaid	__x__None	
11	Stock or stock options	__x__None	
12	Receipt of equipment, materials, drugs, medical writing, gifts or other services	__x__None	
13	Other financial or non-financial interests	__x__None	

Please place an "X" next to the following statement to indicate your agreement:

x___ **I certify that I have answered every question and have not altered the wording of any of the questions on this form.**
